# Economic Burden of Myocardial Infarction Combined With Dyslipidemia

**DOI:** 10.3389/fpubh.2021.648172

**Published:** 2021-02-19

**Authors:** Pingyu Chen, Mengran Zhang, Yan Zhang, Xi Su, Jiyan Chen, Biao Xu, Jianhong Tao, Zhen Wang, Aixia Ma, Hongchao Li

**Affiliations:** ^1^Department of Health Economics, China Pharmaceutical University, Nanjing, China; ^2^Center for Pharmacoeconomics and Outcomes Research, China Pharmaceutical University, Nanjing, China; ^3^Department of Cardiology, Peking University First Hospital, Beijing, China; ^4^Department of Cardiology, Wuhan Asia Heart Hospital, Wuhan, China; ^5^Department of Cardiology, Guangdong Provincial People's Hospital, Guangzhou, China; ^6^Department of Cardiology, Nanjing Gulou Hospital, Nanjing, China; ^7^Department of Cardiology, Sichuan Provincial People's Hospital, Chengdu, China; ^8^Department of Cardiology, Zhongshan Hospital, Fudan University, Shanghai, China

**Keywords:** economic burden, myocardial infarction, dyslipidemia, older adults, China

## Abstract

**Background:** Dyslipidemia is a common comorbidity and an important risk factor for myocardial infarction (MI). This study aimed to examine the economic burden of MI combined with dyslipidemia in China.

**Methods:** Patients who were hospitalized due to MI combined with dyslipidemia in 2016 were enrolled. Costs were measured based on electronic medical records and questionnaires. The annual costs were analyzed by conducting descriptive statistics, univariable, and multivariable analyses.

**Results:** Data of 900 patients were analyzed, and 144 patients were dead during the follow-up. The majority of patients were aged 51–70 years (*n* = 563, 62.55%) and males (*n* = 706, 78.44%). For all-cause costs, the median annual direct medical costs, direct non-medical costs, indirect costs, and total costs were RMB 13,168 (5,212–29,369), RMB 600 (0–1,750), RMB 676 (0–1,787), RMB 15,361 (6,440–33,943), respectively; while for cardiovascular-related costs, the corresponding costs were RMB 12,233 (3,795–23,746), RMB 515 (0–1,680), RMB 587 (0–1,655), and RMB 14,223 (4,914–28,975), respectively. Lifestyle and complications significantly affected both all-cause costs and cardiovascular-related costs.

**Conclusions:** Increasing attention should be paid to encourage healthy lifestyle, and evidence-based medicine should focus on optimal precautions and treatments for complications, to reduce the economic burden among MI patients with a comorbid dyslipidemia.

## Introduction

There are ~290 million cardiovascular patients in China, including 11 million patients with coronary atherosclerotic heart disease, in which myocardial infarction (MI) is the most acute and dangerous disease (1). Cardiovascular diseases have long been the leading cause of death among Chinese residents, while the mortality rate of MI in China had been on the rise from 2002 to 2016, particularly since 2005. In 2016, MI's mortality rate in urban and rural areas reached 58.69 per 100,000 and 74.72 per 100,000 individuals in China, respectively (2).

Dyslipidemia is one of the most significant risk factors for MI. Patients with a history of MI are at high risk and should strictly control their low-density lipoprotein-cholesterol levels (LDL-C) (3, 4). According to the 2002 China Health and Nutrition Survey (CHNS) (5), the 2010 China Chronic Kidney Disease Working Group Survey (6), the 2011 CHNS (7), and the 2015 Report on Nutrition and Chronic Diseases of Chinese residents (8), the prevalence of Dyslipidemia in Chinese people aged ≥18 years in 2002, 2010, 2011, and 2012 were 18.60, 33.97, 39.91, and 40.40%, respectively. In the past 10 years, the prevalence of dyslipidemia as well as MI in Chinese adults have risen sharply. It is estimated that elevated serum cholesterol levels will cause 9.2 million patients with cardiovascular disease (CVD) from 2010 to 2030 (9).

MI, in turn, carries a heavy economic burden. In 2014, the annual direct medical costs of cardiovascular and cerebrovascular diseases in China exceeded 130 billion yuan, accounting for over 22% of the total medical costs in the same year (10). At present, there are few studies directly investigating the economic burden of MI in China. The government bulletin showed that the total and average per hospitalization costs of MI in 2016 were RMB 19.085 billion and RMB 26056.9, respectively, with annual average growth rates of 29.15 and 7.12% (1). The costs of MI hospitalizations increased by 56.8% from 2007 to 2012 in Beijing after adjusting inflation (11). Models predict that universal treatments for hypertension and dyslipidemia patients could avert 10–20 million MIs and between 3 and 10 million CVD deaths during 2016–2030, producing a positive social value net of healthcare costs high as USD 932 billion (12).

Studies on the burden of disease in China only focus on patients' hospitalization costs. There has been no relevant study on this extremely high-risk population of MI with dyslipidemia. Therefore, this study combined patients' medical records and questionnaires to investigate the economic burden for patients with MI and dyslipidemia in China's real-world environment. Our research filled the literature gap by providing the most recent data on the economic burden of MI combined with dyslipidemia from the social perspective, and the factors that potentially impact economic burden were analyzed. Therefore, our results may highlight the importance of providing prevention measures and management intervention. The up-to-date data may be used as important cost parameters for economic evaluations, such as cost of illness or cost-effectiveness/utility analysis.

## Materials and Methods

### Study Design

This is a multicenter study combining retrospective cohort and cross-sectional research and was conducted from a social perspective. Six tertiary hospitals from major cities—Beijing, Shanghai, Wuhan, Nanjing, Guangzhou, and Chengdu, China were chosen as research centers for data collection.

Data were collected according to medical record review and questionnaires, including demographic characteristics (gender, age, marital status, type of medical insurance, income, etc.), lifestyle (smoking, drinking, diet, exercise, etc.), disease characteristics (complications and the cause of death, etc.), hospitalization costs and costs of outpatient (medical and pharmaceutical expenses, nursing expenses, loss of time and labor of the patients and their families, etc.). As the simplified questionnaires were used for the deceased, information, such as lifestyle, income, accommodation and transportation expenses, and out-of-hospital visits were not collected and not able to be analyzed accordingly. The medical record review and questionnaires were linked by patients' name as well as the unique ID number or medical card number.

For all patients, their first hospitalization from January 1, 2016 to December 31, 2016 was considered as the index event, and their related 1-year post-index data was collected and analyzed. Key information of the data is shown in Table 1, the time frame of all the data was 1-year post-index hospitalization, indicating that the cost of index hospitalization was not included in our study

**Table 1:** Key information about the data.

**Variable**	**Patients**	**Source**
**Patient characteristics**		
Survival situation	S, D	QS
Cause of death	D	QS
Gender	S, D	EMR
Birthday	S, D	EMR
Height (cm)	S, D	EMR
Weight (kg)	S, D	EMR
Medical insurance	S, D	EMR
Medical history	S, D	EMR
Education status	S	QS
Marital status	S	QS
Employment status	S	QS
Income level (RMB)	S	QS
Smoking	S	QS
Drinking	S	QS
Diet (whole grains)	S	QS
Diet (High-fat and high-cholesterol)	S	QS
Sports	S	QS
**Costs (hospitalization and outpatient)**		
Direct medical and pharmaceutical expenses	S, D	EMR
Accommodation and transportation expenses	S	QS
Nursing expenses	S	QS
Loss of time and labor of the patients	S	QS
Loss of time and labor of their companions	S	QS
The income of companions	S	QS
Out-of-hospital visit times and costs^*^	S	QS
Health care products costs	S	QS

*S, survivors; D, deceased; QS, questionnaire survey; EMR, electronic medical records*.

* *Costs incurred in other hospitals and pharmacies*.

The study was approved by the Ethics Committees of all the six participating hospitals. Written informed consent was provided by all participants before the data collection and conduction of the research. Before analysis, the patient data were anonymized and de-identified.

### Study Population

Inclusion criteria in this study included: patients hospitalized with myocardial infarction (MI) from January 1, 2016 to December 31, 2016. The earliest hospitalization was considered as the index hospitalization; patients for whom in the first blood lipid examination of the index hospitalization, the LDL-C was ≥1.8 mmol/L or were using lipid-regulating drugs. Exclusion criteria included: patients who died during the index hospitalization; patients who participated in clinical trials after the index hospitalization; patients or family members (if the patient had died) who were not willing to participate in survey or could not correctly understand and answer relevant questions due to communication barriers.

### Outcomes

This study's primary outcomes were the annual total costs of survivors and deceased, which were reported in all-cause and CV-related costs, as well as the direct medical, direct non-medical, and indirect costs. Here CV-related indicates cardiovascular diseases, including MI, hypertension, post-percutaneous transluminal coronary intervention (PCI), peripheral arterial disease, etc. The secondary outcomes were the influencing factors for the annual total all-cause and CV-related costs of survivors.

Direct medical costs included registration, diagnosis, examination, medicine (including pharmacy purchase), operation, bed fee, material, nursing, and other expenses. The direct medical costs were obtained through electronic medical records. Out-of-hospital direct medical costs of the survivors were obtained through questionnaires, while the deceased's out-of-hospital direct medical costs were not collected.

Direct non-medical costs included expenses related to patient caregivers, accommodation and transportation, and health care products, etc. The survivors' data were collected through questionnaires, while the data of the deceased were not collected.

Indirect costs were calculated by the human capital method (13) to calculate the time and labor loss of the patients and their accompanying family members. The monthly income (sum of the pension and additional monthly income for retirees, sum of the monthly salary and extra monthly income for others) of the survivors was derived from the questionnaires. Such data were also not collected for the deceased.

### Statistical Analysis

Described for the baseline characteristics and costs by survival status. Categorical variables were reported as numbers and proportions, and continuous variables were reported as medians and interquartile ranges.

Univariable and multivariable analyses were applied to the study of the economic burden's influencing factors. In the univariable analysis, the chi-square test was performed for categorical variables; the Wilcoxon rank-sum test or the Kruskal-Wallis test was used for continuous variables.

Multivariable analysis was performed in generalized linear model (GLM) with gamma distribution and log link function. Multivariable analysis was not performed for the deceased's total annual costs due to the lack of certain baseline information. Patients' baseline information (age, gender, etc.), lifestyle (smoking, drinking, etc.), and disease history (whether first-episode MI, etc.) were included as independent variables in GLM. The annual all-cause and CV-related total costs of survivors were used as dependent variables.

We combined some variables' classifications in the multivariable analysis since some variables had too many original categories. (a) Medical insurance types: BMISUE, BMISUR, and NRCMS were combined into one category (basic medical insurance). (b) Education status: not graduated from primary school, primary school were combined into primary education; Junior high school, high school, technical secondary school/junior college graduate were combined into medium education; Bachelor, master and doctor were combined into high education. (c) Marital status: unmarried, divorce, and death of a spouse were combined into current single. (d) Employment status: formal employees, individuals, and freelancers were combined into formal wage; Farming, unemployed were combined into non-formal wage.

The significance level was set at *P* < 0.05 with a two-tailed test. Data were analyzed using Stata SE 15 (Stata Software, StataCorp) and SPSS 22.0 (SPSS Software, IBM Corp.).

## Results

### Patient Characteristics

The baseline information of patients is shown in Tables 2, 3. A total of 900 patients were included in this study, including 756 (84.00%) survivors and 144 (16.00%) deceased. Among the deceased, 109 (75.69%) patients died due to CV-related diseases.

**Table 2:** Baseline information of all patients (*N* = 900).

**Variable**	**Number of cases**	**%**
**Survival situation**		
Survival	756	84.00
Death	144	16.00
**Cause of death (*****N*** **= 144)**		
Non-cardiovascular causes	35	24.31
Cardiovascular causes	109	75.69
**Gender**		
Female	194	21.56
Male	706	78.44
Age^*^	62.07 (SD:11.45)	
**Age classification (year)**		
18–50	127	14.11
51–60	256	28.44
61–70	307	34.11
70–	210	23.33
Height (cm)^*^	166.57 (SD:7.04)	
Weight (kg)^*^	69.39 (SD:11.77)	
**BMI (kg/m^2^)**		
−18.4	22	2.44
18.5–23.9	294	32.67
24.0–27.9	450	50.00
28.0–	134	14.89
**Medical insurance**		
Basic medical insurance system for urban employees	584	64.89
Basic medical insurance for urban residents	122	13.56
The new rural cooperative medical care system	89	9.89
Full public expense coverage	42	4.67
Uninsured	53	5.89
Other	8	0.89
**Medical history**		
Myocardial infarction (MI)^#^	48	5.33
Hypertension	537	59.67
Type 2 diabetes	276	30.67
Disorder of lipid metabolism	269	29.89
Post-PCI	582	64.67
Peripheral artery disease	118	13.11

*PCI, percutaneous coronary intervention; BMI, Body mass index*.

* *Mean and standard deviation were reported*.

# *Patients who experienced MI before index hospitalization*.

**Table 3:** Baseline personal and lifestyle information for surviving patients (*N* = 756).

**Variable**	**Number of cases**	**%**
**Education status**		
Not graduated from primary school	51	6.75
Primary school	53	7.01
Junior high school	220	29.10
High school	176	23.28
Technical secondary school/junior college graduate	164	21.69
Bachelor	80	10.58
Master and doctor	11	1.46
**Marital status**		
Unmarried	7	0.93
Married	712	94.18
Divorce	12	1.59
Death of a spouse	25	3.31
**Employment status**		
Formal employees	104	13.76
Individuals and freelancers	40	5.29
Retired	503	66.53
Farming	31	4.10
Unemployed	60	7.94
Other	18	2.38
Income level (RMB)^*^		
0–2,400	187	24.74
2,401–4,000	186	24.60
4,001–5,500	191	25.26
5,501–	192	25.40
**Smoking**		
No smoking history/Give up smoking	496	65.61
Sometimes	88	11.64
Often	172	22.75
**Drinking**		
No drinking history/Give up drinking	515	68.12
Sometimes	186	24.60
Often	55	7.28
**Diet (whole grains)**		
Often	534	70.63
Sometimes	214	28.31
Never	8	1.06
**Diet (high-fat and high-cholesterol)**		
Often control	550	72.75
Sometimes control	170	22.49
Never control	36	4.76
**Sports**		
Often	467	61.77
Sometimes	178	23.54
Never	111	14.68

* *Income is grouped according to quartile*.

There were more males (*n* = 706, 78.44%) than females (*n* = 194, 21.56%). The average age of all patients was 62.07 years (SD: 11.45), and patients aged 60–70 years were the majority (*n* = 307, 34.11%). In terms of body mass index (BMI), most patients (*n* = 450, 50.00%) were overweight (23.9 < BMI ≤ 27.9) or obese (BMI > 27.9) (*n* = 134, 14.89%). Regarding the medical history, 94.67% (*n* = 852) patients were of first-episode MI, 64.67% (*n* = 582) had undergone PCI, and most complications were hypertension (*n* = 537, 59.67%), type 2 diabetes (*n* = 276, 30.67%), and lipid metabolism disorder (*n* = 269, 29.89%). For the type of medical insurance, 88.33% (*n* = 795) had one of the following basic medical insurance which covered majority people in China: basic medical insurance system for urban employees (BMISUE), basic medical insurance system for urban residents (BMISUR), and the new rural cooperative medical system (NRCMS).

Among the 756 surviving patients, the majority (*n* = 560, 74.07%) were of moderate degree (middle-school or equivalent), most (*n* = 712, 94.18%) were married, up to 66.53% (*n* = 503) were retirees, 49.86% had income more than RMB 2,400 and less than RMB 5,500. In terms of lifestyle, the majority had no history of smoking (*n* = 496, 65.61%) or drinking (*n* = 515, 68.12%), often ate high-fiber foods and grains (*n* = 534, 70.63%) and controlled high-fat and high-cholesterol food intake (*n* = 550, 72.75%); 61.77% (*n* = 467) often exercised.

### Total Annual Economic Burden

Table 4 reports the median annual economic burden of the patients. For the all-cause economic burden, the median annual hospitalization costs, outpatient costs, and annual costs for all patients were RMB 0 (IQR: 0–12,756), RMB 10,185 (IQR: 3,300–17,141), and RMB 15,361 (IQR: 6,440–33,943), respectively. The corresponding average annual costs were RMB 18,641 (SD: 45,429), RMB 14,698 (SD: 23602), and RMB 33,878 (SD: 54,029), respectively.

**Table 4:** Median annual economic burden.

**Variable**	**Survivors (*n* = 756)**	**Deceased (*n* = 144)**	**Total (*n* = 900)**
**All-cause**			
**Hospitalization**			
Direct medical costs	0 (0–9,885)	0 (0–12,990)	0 (0–10,000)
Direct non-medical costs	0 (0–600)	NA	0 (0–600)
Indirect costs	0 (0–213)	NA	0 (0–213)
Total cost of hospitalization	0 (0–12,756)	0 (0–12,990)	0 (0–12,756)
**Outpatient**			
Direct medical costs	10,095 (4,143–15,141)	1,142 (0–6,443)	9,006 (2,717–14,565)
Direct non-medical costs	120 (0–600)	NA	120 (0–600)
Indirect costs	423 (0–1,120)	NA	423 (0–1,120)
Total cost of outpatient and emergency	12,146 (5,080–17,997)	1,142 (0–6,443)	10,185 (3,300–17,141)
**Total**			
Total direct medical costs	13,829 (7,492–29,865)	2,547 (0–21,085)	13,168 (5,212–29,369)
Total direct non-medical costs	600 (0–1,750)	NA	600 (0–1,750)
Total indirect costs	676 (0–1,787)	NA	676 (0–1,787)
Total costs	16,737 (9,224–35,025)	2,547 (0–21,085)	15,361 (6,440–33,943)
**Cardiovascular-related**			
**Hospitalization**			
Direct medical costs	0 (0–8,000)	0 (0–0)	0 (0–7,460)
Direct non-medical costs	0 (0–500)	NA	0 (0–500)
Indirect costs	0 (0–0)	NA	0 (0–0)
Total cost of hospitalization	0 (0–10,764)	0 (0–0)	0 (0–10,063)
**Outpatient**			
Direct medical costs	9,674 (3,383–14,469)	888 (0–5,164)	8,014 (2,257–13,813)
Direct non-medical costs	90 (0–600)	NA	90 (0–600)
Indirect costs	373 (0–1,013)	NA	373 (0–1,013)
Total cost of outpatient and emergency	11,127 (4,083–17,150)	888 (0–5,164)	9,341 (2,744–16,208)
**Total**			
Total direct medical costs	13,073 (5,977–25,439)	1,960 (0–14,791)	12,233 (3,795–23,746)
Total direct non-medical costs	515 (0–1,680)	NA	515 (0–1,680)
Total indirect costs	587 (0–1,655)	NA	587 (0–1,655)
Total costs	15,314 (7,766–31,682)	1,960 (0–14,791)	14,223 (4,914–28,975)

*NA, Not applicable (Data for the deceased was not collected)*.

*All the costs were reported as medians and interquartile ranges*.

For the CV-related economic burden, the median annual hospitalization costs, outpatient costs, and annual costs for all patients RMB 0 (IQR: 0–10,063), RMB 9,341 (IQR: 2,744–16,208), RMB 14,223 (IQR: 4,914–28,975). The corresponding average annual costs were RMB 15,480 (SD: 37,233), RMB 12,759 (SD: 19,044), and RMB 28,777 (SD: 43,390), respectively.

The median annual ratio of CV-related costs to all-cause costs was high. The direct medical costs and the hospitalization costs accounted for the largest proportion of total costs.

### Univariable Analysis

The univariable analysis results of the annual all-cause and CV-related costs of survivors are shown in Supplementary Table 1. The corresponding results of the deceased are shown in Supplementary Table 2.

For the survivors, there were significant differences in the annual all-cause costs among patients with different age groups (*P* = 0.049), employment status (*P* = 0.023), income levels (*P* = 0.036), smoking status (*P* = 0.016), drinking status (*P* = 0.016), and dietary control conditions (*P* = 0.020). Complications, such as hypertension (*P* = 0.005), type 2 diabetes (*P* = 0.000), and PCI (*P* = 0.033) significantly affected the annual all-cause total costs. Regarding CV-related costs, patients with different smoking status (*P* = 0.009) and complications including hypertension (*P* = 0.003) and type 2 diabetes (*P* = 0.014) had significantly different costs.

For the deceased, there were significant differences in the annual all-cause costs among patients with different causes of death (*P* = 0.021), BMI (*P* = 0.049), and medical insurance type (*P* = 0.041). In addition, substantial differences existed in the annual CV-related costs among patients with different BMIs (*P* = 0.019) and lipid metabolism disorders (*P* = 0.049).

### Multivariable Analysis

In the GLM model of annual all-cause total costs, 743 patients whose total costs were not 0 without missing categorical variable data were included. In the GLM model of annual CV-related total costs, 741 patients whose total costs were not 0 without missing categorical variable data were included. In the goodness of fit test of the models, the significance levels calculated by the Deviance test and Pearson Chi-Square test were respectively 1.188 and 1.446 for the all-cause model, 1.277 and 1.415 for the CV-related model, none of which were significant (*P* > 0.05), indicating that the two GLM models fit well.

The multivariable analysis results for annual all-cause and CV-related total costs of the survivors are shown in Tables 5, 6. For annual all-cause costs, patients whose income per month was more than RMB 2,400 and less than RMB 4,000 (*P* = 0.003), and was more than RMB 5,500 (*P* = 0.005), who sometimes did sports (*P* = 0.030) had significantly higher total costs. Having type 2 diabetes (*P* = 0.012) was also influencing factors that significantly increased the all-cause total costs. Patients who sometimes (*P* = 0.002) and often (*P* = 0.047) smoke, sometimes control high-fat and high-cholesterol diet (*P* = 0.016) had significantly lower total costs. Having PCI (*P* = 0.000) was also influencing factors that significantly decreased the all-cause total costs.

**Table 5:** Multivariate analysis results of all-cause economic burden on survivors.

**Variable**		**Costs^#^**	**Coef**.	***P*-value**
Gender	Male	18,693 (9,425–43,559)	0	
	Female	16,404 (9,102–34,592)	−0.110	0.411
Age classification (year)	18–50	15041 (6,599–23,610)	0	
	51–60	16,710 (8,577–3,149)	0.092	0.552
	61–70	17,344 (9,920–46,418)	0.264	0.136
	70–	18,682 (12,148–34,592)	−0.024	0.901
BMI (kg/m^2^)	−18.4	15,007 (4,105–20,191)	0	
	18.5–23.9	16,177 (9,123–43,283)	0.250	0.400
	24.0–27.9	16,912 (8,988–34,770)	0.438	0.135
	28.0–	15,622 (9,425–27,838)	0.163	0.604
Medical insurance	Basic medical insurance	16,712 (9,287–34,837)	0	
	Full public expense coverage	25,998 (13,336–59,013)	0.107	0.643
	Uninsured	15,142 (4,153–21,459)	−0.343	0.128
	Other	13,131 (6,303–29,761)	−0.447	0.315
Education status	Primary education	14,294 (9,887–24,162)	0	
	Medium education	16,851 (9,322–37,300)	0.149	0.328
	High education	17,541 (6,909–37,874)	0.200	0.355
Marital status	Current single	21,626 (10,719–55,221)	0	
	Married	16,603 (9,067–34,542)	−0.361	0.070
Employment status	Formal wage	15,641 (7,243–26,712)	0	
	Non-formal wage	17,619 (10,179–42,556)	0.081	0.617
	Retired	14,884 (9,385–28,025)	0.336	0.126
	Other	10,405 (5,557–16,530)	−0.243	0.433
Income level (RMB)	0–2,400	6,451 (13,941–23,145)	0	
	2,401–4,000	10,588 (18,067–54,158)	0.452	0.003^**^
	4,001–5,500	10,083 (16,504–31,846)	0.297	0.059
	5,501–	10,771 (20,076–38,021)	0.477	0.005^**^
**Medical history**				
Myocardial infarction (MI)	No	16,782 (9,403–35,392)	0	
	Yes	15,628 (5,427–31,846)	−0.194	0.362
Hypertension	No	14,884 (6,528–31,846)	0	
	Yes	17,619 (10,818–36,334)	0.077	0.415
Type 2 diabetes	No	15,221 (8,455–30,050)	0	
	Yes	20,355 (11,110–55,567)	0.259	0.012^*^
Disorder of lipid metabolism	No	16,124 (8,867–37,586)	0	
	Yes	17,711 (9,524–30,688)	−0.056	0.585
Post-PCI	No	16,910 (9,042–64,246)	0	
	Yes	16,664 (9,258–29,721)	−0.429	0.000^**^
Peripheral artery disease	No	16,830 (9,219–35,862)	0	
	Yes	15,587 (9,008–31,984)	−0.104	0.447
Smoking	No smoking history/Give up smoking	17,823 (10,131–42,575)	0	
	Sometimes	14,768 (6,002–21,348)	−0.465	0.002^**^
	Often	14,751 (8,098–27,551)	−0.244	0.047^*^
Drinking	No drinking history/Give up drinking	17,418 (9,425–43,559)	0	
	Sometimes	16,734 (8,331–28,395)	−0.206	0.065
	Often	13,628 (5,883–21,897)	−0.293	0.116
Diet (whole grains)	Often	17,485 (8,988–34,770)	0	
	Sometimes	15,304 (9,553–36,038)	−0.032	0.771
	Never	19,268 (7,859–49,900)	−0.313	0.489
Diet (High-fat and high-cholesterol)	Often control	17,823 (9,954–37,874)	0	
	Sometimes control	14,223 (7,286–25,787)	−0.282	0.016^*^
	Never control	16,568 (7,087–42,316)	−0.134	0.549
Sports	Often	15,978 (7,866–31,846)	0	
	Sometimes	16,877 (9,789–36,038)	0.254	0.030^*^
	Never	18,595 (10,933–51,003)	0.228	0.091

*PCI, percutaneous coronary intervention; BMI, body mass index*.

* *p < 0.05*,

** *p < 0.01*.

# *All the costs were reported as medians and interquartile ranges*.

**Table 6:** Multivariate analysis results of cardiovascular-related economic burden on survivors.

**Variable**		**Costs^#^**	**Coef**.	***P*-value**
Gender	Male	17,551 (8,000–33,701)	0	
	Female	15,107 (7,661–30,342)	−0.146	0.270
Age classification (year)	18–50	15,041 (6,200–23,140)	0	
	51–60	16,535 (7,830–30,342)	0.109	0.475
	61–70	15,378 (8,362–35,538)	0.204	0.242
	70–	15,085 (8,168–34,492)	−0.083	0.666
BMI (kg/m^2^)	−18.4	9,017 (3,867–18,028)	0	
	18.5–23.9	14,693 (7,830–35,685)	0.216	0.464
	24.0–27.9	16,517 (7,174–32,058)	0.361	0.213
	28.0–	14,885 (9,258–27,838)	0.111	0.720
Medical insurance	Basic medical insurance	15,251 (8,029–32,128)	0	
	Full public expense coverage	16,944 (7,801–35,685)	−0.354	0.119
	Uninsured	12,305 (4,153–20,637)	−0.350	0.115
	Other	13,131 (6,250–29,761)	−0.430	0.330
Education status	Primary education	14,227 (8,443–24,162)	0	
	Medium education	15,251 (7,848–32,032)	0.088	0.564
	High education	16,878 (6,200–35,685)	0.197	0.362
Marital status	Current single	18,044 (9,635–44,311)	0	
	Married	15,219 (7,414–30,372)	−0.287	0.146
Employment status	Formal wage	15,467 (5,325–25,396)	0	
	Non-formal wage	15,824 (7,925–34,605)	0.047	0.770
	Retired	15,226 (8,510–32,755)	0.393	0.071
	Other	10,405 (5,557–16,530)	−0.247	0.421
Income level (RMB)	0–2,400	5,145 (13,570–23,145)	0	
	2,401–4,000	10,270 (18,067–49,722)	0.457	0.002^**^
	4,001–5,500	8,331 (14,283–27,103)	0.277	0.077
	5,501–	7,947 (17,984–34,472)	0.440	0.010^*^
**Medical history**				
Myocardial infarction (MI)	No	15,557 (7,925–32,058)	0	
	Yes	12,614 (4,854–22,399)	−0.419	0.045^*^
Hypertension	No	13,747 (5,285–28,000)	0	
	Yes	16,732 (9,552–33,771)	0.119	0.202
Type 2 diabetes	No	14,652 (6,909–28,395)	0	
	Yes	17,637 (8,577–41,649)	0.261	0.012^*^
Disorder of lipid metabolism	No	15,358 (7,245–34,229)	0	
	Yes	15,159 (8,343–27,147)	−0.061	0.551
Post-PCI	No	15,598 (7,308–50,294)	0	
	Yes	15,096 (8,000–27,758)	−0.358	0.001^**^
Peripheral artery disease	No	15,634 (7,848–32,322)	0	
	Yes	14,321 (6,390–24,979)	−0.117	0.391
Smoking	No smoking history/Give up smoking	16,207 (8,673–37,102)	0	
	Sometimes	14,436 (5,177–21,418)	−0.452	0.003^**^
	Often	14,275 (7,312–23,751)	−0.345	0.005^**^
Drinking	No drinking history/Give up drinking	15,641 (8,093–36,906)	0	
	Sometimes	15,901 (7,801–26,541)	−0.133	0.230
	Often	12,788 (5,883–21,897)	−0.219	0.234
Diet (whole grains)	Often	15,218 (7,061–30,050)	0	
	Sometimes	15,495 (9,123–34,598)	−0.028	0.793
	Never	19,268 (7,579–49,900)	−0.217	0.628
Diet (High-fat and high-cholesterol)	Often control	15,962 (8,058–33,771)	0	
	Sometimes control	14,223 (6,611–25,787)	−0.211	0.066
	Never control	16,187 (5,978–36,755)	−0.024	0.916
Sports	Often	14,717 (6,546–29,253)	0	
	Sometimes	16,686 (9,316–34,605)	0.241	0.036^*^
	Never	16,081 (9,524–39,809)	0.164	0.220

*PCI, percutaneous coronary intervention; BMI, body mass index*.

* *p < 0.05*,

** *p < 0.01*.

# *All the costs were reported as medians and interquartile ranges*.

While for annual CV-related costs, patients with basic medical insurance, patients whose income per month was more than RMB 2,400 and less than RMB 4,000 (*P* = 0.002), and was more than RMB 5,500 (*P* = 0.010), who sometimes did sports (*P* = 0.036) had significantly higher total costs. Having type 2 diabetes (*P* = 0.012) was also influencing factors that significantly increased the all-cause total costs. Patients who sometimes (*P* = 0.003) and often (*P* = 0.005) smoke had significantly lower total costs. Having MI history (*P* = 0.045) and PCI (*P* = 0.012) was also influencing factors that significantly decreased the all-cause total costs.

## Discussion

MI is a public health challenge in China and throughout the world. Dyslipidemia is one of the main risk factors for MI, and it also brings a tremendous economic burden on society. However, unlike some European and American countries, no Asian countries, including China, have measured patients' economic burden with MI combined with dyslipidemia. Our investigation is the first study to investigate the costs of patients with MI combined with dyslipidemia in China from the whole society's perspective using the real-world data in China.

In our study, we reported the median annual all-cause and CV-related costs, related studies were very limited. The hospitalization costs per time of patients with MI in Beijing in 2012 were RMB 29,000 (11), which was slightly higher than that (RMB 23,690 in 2017) in our study. Compared with patients' economic burden with MI in other countries (14–16), our study's annual all-cause and CV-related costs were lower. It may be related to differences in sample sizes, medical resource utilization, and MI treatment in different countries. Besides, in our study, CV-related costs accounted for a large proportion of the total all-cause costs, indicating that CV-related diseases' economic burden was high.

There were significant differences in both annual all-cause and CV-related total costs in terms of gender, BMI, medical insurance, medical history, and other factors. The average age of all patients in our study was 62.1 years, most of them were between 50 and 70 years and male. The characteristics of gender and age are consistent with other studies that reported baseline information (11, 14, 17). Costs were also high in those older and retired patients, especially for males. It was showed that MI's economic burden of patients >60 years was ~47% of the total costs in 2012 in Korea (18). Older people may have a greater likelihood of suffering from MI (19), may require a more extended hospital stay to achieve the treatment effect (19), and maybe more likely to have other complications (19), resulting in higher costs.

The all-cause and CV-related total costs were significantly higher among patients with type 2 diabetes. The costs of treatment for the disease, the multiple hospital prescriptions required for chronic disease, and the disease progression of MI due to type 2 diabetes (20–22) may result in larger numbers and costs for MI hospitalizations. Patients who had been subjected to PCI had lower total costs, and patients with first-episode MI had higher total CV-related costs. It may be because PCI's efficacy (23, 24) reduces the number of required hospital stay and visits, patients with a first-episode MI need to be more vigilant about their health.

Smoking and diet did not affect all-cause and CV-related total costs of survivors in an expected way. Some studies (25, 26) have shown that never or quitting smoking positively affects the number and risk of hospitalization for MI. The reduction of smoking prevalence decreased the direct medical costs and the mortality of MI that occur before people went to the hospital (27). However, Yegezu et al. (28) observed that survivors with MI were more likely to be smoking patients, and a healthy diet could reduce fat intake, reducing the risk of dyslipidemia. Research showed that the Mediterranean diet reduced cardiovascular death, complications, and hospital admissions of MI (29). In our study, dietary factors had a different impact on the costs than those reported in the above research. The reason may be that patients with high-fat and high-cholesterol intake may also exercise more, resulting in normal blood lipid levels (30, 31). When fat loss from exercise is more than fat intake, the risk of dyslipidemia will be reduced, decreasing costs. More research is needed on the effects of smoking and diet on hospitalization costs and outpatient costs among patients with MI.

The strengths of this study lie in the following. First, from the perspective of target groups, our study is the first to consider the costs of Chinese patients with MI combined with dyslipidemia. Second, from the standpoint of data sources, the study used patients' electronic medical record data as the primary source of real-world data, supplemented by questionnaires. Compared with entirely relying on questionnaires and the patients' subjective recall, the data are more reliable. Third, in terms of costs, compared with similar studies that only consider the direct medical costs or hospitalization costs, the types of costs included in this research are more comprehensive (direct medical costs, direct non-medical costs, and indirect costs). The median annual all-cause and CV-related costs of hospitalization and outpatient of patients are reported, allowing readers to understand the economic burden of this population multi-dimensionally.

Our study also has some limitations. First, data on direct non-medical costs and indirect costs were derived from questionnaires, which can be more subjective than written or electronically recorded data. Second, specific costs regarding medical insurance reimbursement and out-of-pocket were missing; therefore, it was impossible to compare and analyze the out-of-pocket and medical insurance fund expenditures in this study.

## Conclusions

Chinese patients with MI combined with dyslipidemia have high all-cause and CV-related costs, among which hospitalization and direct medical costs account for a large proportion. Our findings suggest that a healthy lifestyle contributes a lot and optimal precautions and treatments for complications should be emphasized, which can help to reduce the economic burden. It is also anticipated that the descriptive annual costs will act as a significant new resource for the cost-of-illness studies as well as economic evaluations regarding this population.

## Data Availability Statement

The datasets generated for this article are not readily available because sharing of raw data would be contingent on approval from the research ethics office because of the ethics consideration. Requests to access the datasets should be directed to lihongchao@cpu.edu.cn.

## Ethics Statement

The studies involving human participants were reviewed and approved by Peking University First Hospital; Wuhan Asia Heart Hospital; Guangdong Provincial People's Hospital; Nanjing Gulou Hospital; Sichuan Provincial People's Hospital; Zhongshan Hospital, Fudan University. The patients/participants provided their written informed consent to participate in this study.

## Author Contributions

HL, AM, and PC designed this study. Data were collected and managed by YZ, XS, JC, BX, JT, and ZW. PC and MZ performed the statistics using software and drafted the manuscript. HL and AM supervised the data analysis and proposed suggestions for revising the manuscript. All authors critically reviewed the manuscript, approved the final version of the manuscript, and agreed to be accountable for the content of the work.

## Conflict of Interest

Amgen Biotech Consultation (Shanghai) Co., Ltd partly sponsored this study. However, this study is an economic burden investigation, which does not involve any specific products. Therefore, the authors declare that the research was conducted in the absence of any commercial or financial relationships that could be construed as a potential conflict of interest.
