# Supplementary material for: Economic Burden of Myocardial Infarction Combined With Dyslipidemia
Source: Front Public Health. 2021 Feb 19;9:648172. doi: 10.3389/fpubh.2021.648172 (PMC7933193; doi:10.3389/fpubh.2021.648172)
Supplement: Supplementary file 1 [file Data_Sheet_1.docx]

Supplementary Material

**Supplementary Table 1.** Univariate analysis of the economic burden on survivors

| **Variable** | **All-cause costs median (IQR)** | ***P* value** | **Cardiovascular-related costs median (IQR)** | ***P* value** |
| --- | --- | --- | --- | --- |
| Gender |  | 0.474 |  | 0.493 |
| Female | 18,693 (9,425-43,559) |  | 17,551 (8,000-33,701) |  |
| Male | 16,404 (9,102-34,592) |  | 15,107 (7,661-30,342) |  |
| Age classification (year) |  | 0.049* |  | 0.440 |
| 18-50 | 15041 (6,599-23,610) |  | 15,041 (6,200-23,140) |  |
| 51-60 | 16,710 (8,577-3,149) |  | 16,535 (7,830-30,342) |  |
| 61-70 | 17,344 (9,920-46,418) |  | 15,378 (8,362-35,538) |  |
| 70- | 18,682 (12,148-34,592) |  | 15,085 (8,168-34,492) |  |
| BMI (kg/m^2^) |  | 0.611 |  | 0.301 |
| -18.4 | 15,007 (4,105-20,191) |  | 9,017 (3,867-18,028) |  |
| 18.5-23.9 | 16,177 (9,123-43,283) |  | 14,693 (7,830-35,685) |  |
| 24.0-27.9 | 16,912 (8,988-34,770) |  | 16,517 (7,174-32,058) |  |
| 28.0- | 15,622 (9,425-27,838) |  | 14,885 (9,258-27,838) |  |
| Medical insurance |  | 0.057 |  | 0.460 |
| Basic medical insurance system for urban employees | 16,851 (9,329-34,685) |  | 15,314 (8,093-31,865) |  |
| Basic medical insurance for urban residents | 17,617 (9,425-39,809) |  | 16,881 (8,058-32,755) |  |
| The new rural cooperative medical care system | 14,267 (8,471-29,453) |  | 14,883 (7,308-29,453) |  |
| Full public expense coverage | 25,998 (13,336-59,014) |  | 16,944 (7,801-35,685) |  |
| Uninsured | 15,142 (4,153-21,459) |  | 12,305 (4,153-20,637) |  |
| Other | 13,131 (6,303-29,761) |  | 13,131 (6,250-29,761) |  |
| Education status |  | 0.151 |  | 0.178 |
| Not graduated from primary school | 14,220 (9,887-22,894) |  | 14,220 (8,443-23,145) |  |
| Primary school | 14,294 (9,258-30,050) |  | 14,227 (8,058-24,162) |  |
| Junior high school | 15,242 (8,393-27,596) |  | 14,423 (6,499-25,367) |  |
| High school | 15,934 (77,456-37,835) |  | 16,572 (7,746-36,720) |  |
| Technical secondary school/junior college graduate | 19,646 (12,144-47,251) |  | 17,206 (10,190-38,965) |  |
| Bachelor | 17,417 (7,440-36,780) |  | 16,987 (6,317-36,296) |  |
| Master and doctor | 17,541 (5,085-38,867) |  | 13,747 (3,609-26,635) |  |
| Marital status |  | 0.211 |  | 0.237 |
| Unmarried | 29,453 (24,737-44,620) |  | 29,453 (12,507-44,620) |  |
| Married | 16,603 (9,067-34,542) |  | 15,219 (7,414-30,372) |  |
| Divorce | 14,130 (10,259-20,391) |  | 12,291 (6,120-17,251) |  |
| Death of a spouse | 22,548 (10,862-80,075) |  | 22,548 (9,329-64,173) |  |
| Employment status |  | 0.023* |  | 0.120 |
| Formal employees | 15,641 (7,243-29,148) |  | 15,677 (6,421-27,993) |  |
| Individuals and freelancers | 16,139 (6,916-23,375) |  | 14,153 (4,047-22,745) |  |
| Retired | 17,619 (10,179-42,556) |  | 15,824 (7,925-34,605) |  |
| Farming | 14,884 (10,355-38,209) |  | 19,057 (12,257-50,294) |  |
| Unemployed | 15,055 (8,884-21,353) |  | 15,055 (8,029-21,026) |  |
| Other | 10,405 (5,557-16,530) |  | 10,405 (5,557-16,530) |  |
| Income level (RMB) |  | 0.002** |  | 0.002** |
| 0-2400 | 6,451 (13,941-23,145) |  | 5,145 (13,570-23,145) |  |
| 2401-4000 | 10,588 (18,067-54,158) |  | 10,270 (18,067-49,722) |  |
| 4001-5500 | 10,083 (16,504-31,846) |  | 8,331 (14,283-27,103) |  |
| 5501- | 10,771 (20,076-38,021) |  | 7,947 (17,984-34,472) |  |
| Medical history |  |  |  |  |
| Myocardial infarction (MI) |  | 0.186 |  | 0.074 |
| No | 16,782 (9,403-35,392) |  | 15,557 (7,925-32,058) |  |
| Yes | 15,628 (5,427-31,846) |  | 12,614 (4,854-22,399) |  |
| Hypertension |  | 0.005** |  | 0.003** |
| No | 14,884 (6,528-31,846) |  | 13,747 (5,285-28,000) |  |
| Yes | 17,619 (10,818-36,334) |  | 16,732 (9,552-33,771) |  |
| Type 2 diabetes |  | 0.000** |  | 0.014* |
| No | 15,221 (8,455-30,050) |  | 14,652 (6,909-28,395) |  |
| Yes | 20,355 (11,110-55,567) |  | 17,637 (8,577-41,649) |  |
| Disorder of lipid metabolism |  | 0.754 |  | 0.575 |
| No | 16,124 (8,867-37,586) |  | 15,358 (7,245-34,229) |  |
| Yes | 17,711 (9,524-30,688) |  | 15,159 (8,343-27,147) |  |
| Post PCI |  | 0.033* |  | 0.113 |
| No | 16,910 (9,042-64,246) |  | 15,598 (7,308-50,294) |  |
| Yes | 16,664 (9,258-29,721) |  | 15,096 (8,000-27,758) |  |
| Peripheral artery disease |  | 0.556 |  | 0.195 |
| No | 16,830 (9,219-35,862) |  | 15,634 (7,848-32,322) |  |
| Yes | 15,587 (9,008-31,984) |  | 14,321 (6,390-24,979) |  |
| Smoking |  | 0.016* |  | 0.009** |
| No smoking history /Give up smoking | 17,823 (10,131-42,575) |  | 16,207 (8,673-37,102) |  |
| Sometimes | 14,768 (6,002-21,348) |  | 14,436 (5,177-21,418) |  |
| Often | 14,751 (8,098-27,551) |  | 14,275 (7,312-23,751) |  |
| Drinking |  | 0.016* |  | 0.115 |
| No drinking history /Give up drinking | 17,418 (9,425-43,559) |  | 15,641 (8,093-36,906) |  |
| Sometimes | 16,734 (8,331-28,395) |  | 15,901 (7,801-26,541) |  |
| Often | 13,628 (5,883-21,897) |  | 12,788 (5,883-21,897) |  |
| Diet (Whole Grains) |  | 0.817 |  | 0.718 |
| Often | 17,485 (8,988-34,770) |  | 15,218 (7,061-30,050) |  |
| Sometimes | 15,304 (9,553-36,038) |  | 15,495 (9,123-34,598) |  |
| Never | 19,268 (7,859-49,900) |  | 19,268 (7,579-49,900) |  |
| Diet (High-fat and high-cholesterol) |  | 0.020* |  | 0.306 |
| Often control | 17,823 (9,954-37,874) |  | 15,962 (8,058-33,771) |  |
| Sometimes control | 14,223 (7,286-25,787) |  | 14,223 (6,611-25,787) |  |
| Never control | 16,568 (7,087-42,316) |  | 16,187 (5,978-36,755) |  |
| Sports |  | 0.216 |  | 0.207 |
| Often | 15,978 (7,866-31,846) |  | 14,717 (6,546-29,253) |  |
| Sometimes | 16,877 (9,789-36,038) |  | 16,686 (9,316-34,605) |  |
| Never | 185,95 (10,933-51,003) |  | 16,081 (9,524-39,809) |  |
| PCI: percutaneous coronary intervention; BMI: Body mass index  * p<0.05, ** p<0.01 | | | | |

**Supplementary Table 2.** Univariate analysis of the economic burden on the deceased

| **Variable** | **All-cause costs median (IQR)** | ***P* value** | **Cardiovascular-related costs median (IQR)** | ***P* value** |  |
| --- | --- | --- | --- | --- | --- |
| Cause of death |  | 0.021* |  | 0.059 |  |
| Non-cardiovascular causes | 7,485 (1,844-58,862) |  | 4,362 (854-13,491) |  |  |
| Cardiovascular causes | 1,661 (0-19,194) |  | 1,056 (0-15,668) |  |  |
| Gender |  | 0.585 |  | 0.421 |  |
| Female | 2,406 (0-26,143) |  | 1,283 (0-11,290) |  |  |
| Male | 2,757 (0-19,268) |  | 2,023 (0-16,667) |  |  |
| Age classification (year) |  | 0.724 |  | 0.946 |  |
| 18-50 | 11,639 (1,362-37,318) |  | 2,723 (1,283-19,194) |  |  |
| 51-60 | 1,797 (0-33,631) |  | 1,377 (0-7,373) |  |  |
| 61-70 | 2,504 (0-12,487) |  | 1,844 (0-12,381) |  |  |
| 70- | 2,590 (0-19,887) |  | 2,005 (0-15,668) |  |  |
| BMI (kg/m^2^) |  | 0.049* |  | 0.019* |  |
| -18.4 | 775 (0-16,666) |  | 737 (0-6,740) |  |  |
| 18.5-23.9 | 10,785 (8-37,200) |  | 4,361 (8-30,102) |  |  |
| 24.0-27.9 | 1,142 (0-12,979) |  | 533 (0-6,056) |  |  |
| 28.0- | 7,485 (819-24,678) |  | 2,749 (819-19,194) |  |  |
| Medical insurance |  | 0.041* |  | 0.307 |  |
| Basic medical insurance system for urban employees | 3,277 (8-26,241) |  | 2,674 (0-18,324) |  |  |
| Basic medical insurance for urban residents | 2,278 (0-14,499) |  | 2,278 (0-16,667) |  |  |
| The new rural cooperative medical care system | 309 (0-7,832) |  | 309 (0-2,726) |  |  |
| Uninsured | 35,633 (19,194-103,573) |  | 0 (0-19,194) |  |  |
| Other | 7,013 (0-31,802) |  | 2,386 (0-11,290) |  |  |
| Medical history |  |  |  |  |  |
| Myocardial infarction (MI) |  | 0.291 |  | 0.234 |  |
| No | 2,503 (0-19,268) |  | 1,800 (0-13,249) |  |  |
| Yes | 15,083 (1,228-51,944) |  | 9,481 (1228-19,887) |  |  |
| Hypertension |  | 0.080 |  | 0.135 |  |
| No | 1,511 (0-14,459) |  | 1,069 (0-7,057) |  |  |
| Yes | 4,941 (0-26,381) |  | 2,318 (0-18,897) |  |  |
| Type 2 diabetes |  | 0.477 |  | 0.685 |  |
| No | 2,590 (0-16,667) |  | 2,278 (0-15,668) |  |  |
| Yes | 2,502 (0-37,318) |  | 1,362 (0-12,465) |  |  |
| Disorder of lipid metabolism |  | 0.099 |  | 0.049* |  |
| No | 2,403 (0-19,268) |  | 1,228 (0-12,367) |  |  |
| Yes | 8,568 (819-26,520) |  | 4,463 (819-19,194) |  |  |
| Post PCI |  | 0.110 |  | 0.060 |  |
| No | 1,960 (0-18,796) |  | 1,322 (0-8,313) |  |  |
| Yes | 7,817 (203-32,716) |  | 3,226 (0-22,718) |  |  |
| Peripheral artery disease |  | 0.476 |  | 0.251 |  |
| No | 2,503 (0-19,268) |  | 1,716 (0-13,249) |  |  |
| Yes | 7,479 (0-26,241) |  | 2,884 (0-26,241) |  |  |
| PCI: percutaneous coronary intervention; BMI: Body mass index  * p<0.05, ** p<0.01 | | | | | |
